# AaPDR3, a PDR Transporter 3, Is Involved in Sesquiterpene β-Caryophyllene Transport in *Artemisia annua*

**DOI:** 10.3389/fpls.2017.00723

**Published:** 2017-05-08

**Authors:** Xueqing Fu, Pu Shi, Qian He, Qian Shen, Yueli Tang, Qifang Pan, Yanan Ma, Tingxiang Yan, Minghui Chen, Xiaolong Hao, Pin Liu, Ling Li, Yuliang Wang, Xiaofen Sun, Kexuan Tang

**Affiliations:** Joint International Research Laboratory of Metabolic and Developmental Sciences, Key Laboratory of Urban Agriculture (South) Ministry of Agriculture, Plant Biotechnology Research Center, Fudan-SJTU-Nottingham Plant Biotechnology R&D Center, School of Agriculture and Biology, Shanghai Jiao Tong UniversityShanghai, China

**Keywords:** *Artemisia annua* L., sesquiterpene, ABC transporter, β-caryophyllene, pleiotropic drug resistance (PDR) transporter

## Abstract

Artemisinin, a sesquiterpenoid endoperoxide, isolated from the plant *Artemisia annua* L., is widely used in the treatment of malaria. Another sesquiterpenoid, β-caryophyllene having antibiotic, antioxidant, anticarcinogenic and local anesthetic activities, is also presented in *A. annua*. The role played by sesquiterpene transporters in trichomes and accumulation of these metabolites is poorly understood in *A. annua* and in trichomes of other plant species. We identified *AaPDR3*, encoding a pleiotropic drug resistance (PDR) transporter located to the plasma membrane from *A. annua*. Expression of *AaPDR3* is tissue-specifically and developmentally regulated in *A. annua*. GUS activity is primarily restricted to T-shaped trichomes of old leaves and roots of transgenic *A. annua* plants expressing *proAaPDR3*: *GUS*. The level of β-caryophyllene was decreased in transgenic *A. annua* plants expressing *AaPDR3*-RNAi while transgenic *A. annua* plants expressing increased levels of *AaPDR3* accumulated higher levels of β-caryophyllene. When AaPDR3 was expressed in transformed yeast, yeasts expressing *AaPDR3* accumulated more β-caryophyllene, rather than germacrene D and β-farnesene, compared to the non-expressing control.

## Introduction

ATP-binding-cassette (ABC) proteins are one of the biggest protein families in plants, which function as channels, molecular switches, and transporters (Sugiyama et al., 2006). ABC transporters are divided into different subfamilies depending on the combination of the structural elements (Verrier et al., 2008). One family of these, pleiotropic drug resistance (PDR) transporters, the full size ABCG subfamily, consist of two transmembrane domains (TMDs) and two nucleotide binding domains (NBDs). The NBDs contain Walker A motifs, Walker B motifs, and the ABC signature motifs (Biemansoldehinkel et al., 2006). In plants, PDR transporters are reported to be involved in varieties of biological functions, including terpenoids and phytohormone transport, cuticular formation, defense against pathogens, and resistance to cadmium and lead (Jasiński et al., 2001; Lee et al., 2005; Stukkens et al., 2005; Ito and Gray, 2006; Kobae et al., 2006; Strader and Bartel, 2009; Kang et al., 2010; Kim et al., 2010; Bessire et al., 2011). The first plant PDR transporter, SpTUR2, was cloned from *Spirodela polyrrhiza*, which might play a role in response to conditions inhibiting plant growth (Smart and Fleming, 1996). Then SpTUR2 was conferred on the resistance to the antifungal diterpene sclareol (Van Den Brûle et al., 2002). The work on ABC transporters in *Nicotiana plumbaginifolia* showed that *NpABC1* was regulated by the antifungal diterpenes sclareol and sclareolide in cell cultures (Jasiński et al., 2001). Subsequently NpPDR1 was reported to be involved in the secretion of defense-related metabolites (Stukkens et al., 2005). And the expression of NtPDR1 in *Nicotiana tabacum* BY2 cells and transport tests suggested that NtPDR1 was involved in diterpene transport to defend against biotic threats (Crouzet et al., 2013). Besides, it has been reported that (AtPDR12)/ABCG40 mediates cellular uptake of the phytohormone abscisic acid (Kang et al., 2010). Furthermore, some PDR transporters were reported to contribute to heavy metals resistance, such as cadmium (Cd^2+^) and lead (Pb^2+^) (Lee et al., 2005; Kim et al., 2007). Cadmium and lead are common pollutants in soil, which are dangerous to plants growth (Raskin et al., 1997; Lanphear, 1998). In plants, *AtPDR8*-overexpressing plants showed stronger Cd^2+^ or Pb^2+^ resistance, and *AtPDR8* RNAi transgenic plants and T-DNA insertion lines were more sensitive to Cd^2+^ or Pb^2+^ compared to wild-type plants (Kim et al., 2007). AtPDR12, an ABC transporter, was reported to contribute to Pb^2+^ resistance in *Arabidopsis* (Lee et al., 2005).

*Artemisia annua* L., a traditional Chinese medicinal plant, is famous for producing the sesquiterpenoid endoperoxide artemisinin. Artemisinin-based combination therapies (ACTs) are a recommended treatment against the cerebral and chloroquine-resistant malaria by the World Health Organization (WHO; White, 2008). In addition to artemisinin, a large number of monoterpenes, sesquiterpenes, and triterpenes are presented in *A. annua* with functions in growth, development and defense in plants (Wei et al., 1992; Fulzele et al., 1995; Holm et al., 1997; Tellez et al., 1999; Bhakuni et al., 2001; Goel et al., 2007). In fact, the monoterpenes from *A. annua* contain the regular monoterpenes, the rearranged monoterpenes, and the irregular monoterpenes (Charles et al., 1991; Woerdenbag et al., 1994; Jia et al., 1999). The sesquiterpenes β-caryophyllene, β-farnesene, germacrene D, germacrene A, amorphadiene, and epi-cedrol were isolated from *A. annua* (Fulzele et al., 1995; Bouwmeester et al., 1999; Juteau et al., 2002). Monoterpenes and sesquiterpenes as the major volatile compounds of plants are usually emitted to defend against biotic threats (Degenhardt et al., 2003). For example, (E)-β-farnesene (EβF) is an important volatile compound of plants, which functions as the main component of the aphid alarm pheromones (Bowers et al., 1972; Pickett and Griffiths, 1980; Francis et al., 2004). A sesquiterpene, β-caryophyllene, is distributed in essential oils of plants with the anti-inflammatory, antibiotic, antioxidant, anticarcinogenic, and local anesthetic activities (Legault and Pichette, 2007). The triterpenoids include sterols, steroids, and saponins, are a large and structurally diverse group of natural products, derived from squalene (Xu et al., 2004).

With so many varieties, the sesquiterpene biosynthesis network is quite complicated in *A. annua* (Figure S1). Fortunately, several sesquiterpene synthases have been reported from *A. annua*. Sesquiterpenes, like artemisinin, are synthesized via the direct precursor farnesyl diphosphate (FPP) in plants. In sesquiterpene biosynthesis, FPP is converted to an array of cyclization products, such as amorpha-4,11-diene, β-caryophyllene, β-farnesene, germacrene A, and epi-cedrol, by amorpha-4,11-diene synthase (AaADS; Bouwmeester et al., 1999), β-caryophyllene synthase (AaCPS; Cai et al., 2002), β-farnesene synthase (AaBFS; Picaud et al., 2005), germacrene A synthase (AaGAS; Bertea et al., 2006), and epi-cedrol synthase (AaECS; Mercke et al., 1999) respectively in *A. annua*. In addition, it is well-known that geranyl diphosphate (GPP) is the precursor of monoterpenes. The formation of monoterpene linalool is catalyzed by linalool synthase (AaLAS; Jia et al., 1999). Squalene synthase (AaSQS) is a key enzyme of sterol and triterpene pathway (Liu et al., 2003). The synthesis of triterpene β-Amyrin is catalyzed by β-Amyrin synthase (AaBAS).

There are two kinds of trichomes in *A. annua*, glandular trichomes and T-shaped trichomes, in which large quantities of secondary metabolites are synthetized, stored and volatilized to protect plants against plant pathogens, neighboring plants, insects, and herbivores (Wagner, 1991; Duke and Paul, 1993; Pichersky and Gershenzon, 2002). The glandular trichomes where artemisinin biosynthesis occurs, contains two stalk cells, two basal cells, and three pairs of secretory cells (Duke and Paul, 1993; Olsson et al., 2009). By contrast, the research on T-shaped trichomes is still largely unknown. Previous studies demonstrated that AaCPS was primarily located in T-shaped trichomes, roots, buds, and flowers, while AaBFS was expressed in T-shaped trichomes, glandular trichomes, and roots (Wang et al., 2013, 2014). The transcriptome of T-shaped trichomes was sequenced using Illumina RNA-Seq. The result showed that the specific terpene metabolic pathways were also existed in the T-shaped trichome (Soetaert et al., 2013). In one publication, the authors cloned PDR1 and PDR2 transporters from *A. annua* and suggested that PDR2 was related to artemisinin biosynthesis in tobacco, although the substrate was not verified (Wang et al., 2016).

Therefore, these findings indicate that the multicellular T-shaped trichomes have the capacity to synthesize and store large quantities of sesquiterpenes in *A. annua*. Numerous studies have identified genes related to sesquiterpenes biosynthesis in *A. annua*, but little is known about the sesquiterpenes transport. Hence, it will be interesting to investigate sesquiterpenes transporters in the biofactories. Here, we identified a PDR transporter PDR3 (AaPDR3) from the T-shaped trichomes RNAseq databases, which is specifically expressed and developmentally regulated in *A. annua*. The decrease and increase in the transcript levels of *AaPDR3* in the RNAi and overexpression plants resulted in the decrease and increase of β-caryophyllene contents, respectively. Besides, when *AaPDR3* was expressed in yeast, β-caryophyllene was accumulated faster than the control. From these results, we identified a PDR transporter involved in β-caryophyllene transport in *A. annua*.

## Experimental procedures

### Plant material and growth conditions

*A. annua* named as “Huhao 1,” originated from Chongqing, was developed in Shanghai after selection for several years. Plants were grown in the greenhouse with a 16/8 h light/dark photoperiod at 25°C.

### Isolation and characterization of *AaPDR3*

T-shape trichomes were collected from the capitulum of *A. annua* with laser capture microdissection. The RNA from T-shape trichomes was extracted and sequenced (Soetaert et al., 2013). *Arabidopsis* ABC protein sequences were obtained from the Arabidopsis Information Resource (TAIR) database. *A. annua* putative ABC transporters were searched performing a BLASTP analysis against the transcriptome database using *Arabidopsis* ABC transporter protein sequences as queries with an “E” value over e^−120^. Then the sequences of polypeptides corresponding to *A. annua* ABC transporters were analyzed in the Conserved Domain Database (CDD) at NCBI (Cakir and Kilickaya, 2013). The ABC transporters protein sequences from *A. annua* and PDR protein sequences from *Arabidopsis* were aligned with ClustalX. The phylogenetic tree was constructed by MEGA software (Tamura et al., 2011). Based on the RNAseq databases, we predicted the full-length AaPDR3 sequence. To obtain the open reading frame (ORF) of *AaPDR3*, the cDNA was synthesized with 0.5 μg total RNA isolated from leaves of *A. annua*, and the ORF was amplified using the gene-specific primers (Table S1). The phylogenetic tree analysis was performed with MEGA software version 5 via the neighbor-joining method based on amino acid sequence alignment, and the bootstrap analysis was performed using 1,000 replicates. Roots, stems, young leaves (the two youngest leaves), old leaves (from the 15th to the 16th leaf), buds and flowers of the *A. annua* plants were collected for RNA extraction using plant RNA isolation reagent (Tiangen, Beijing, China) following the manufacturer's instructions. The leaves from the Leaf0 (meristem), Leaf1, Leaf2, Leaf3, Leaf4, Leaf5, and Leaf6 counted from the apical top of the main stem were collected from 5-month-old *A. annua*. The total RNA was used to synthesize the first-strand cDNA. All the tissues and leaves collected from three plants were separately pooled for each determination. For hormone treatment, 2-month-old *A. annua* plants were treated with 100 μM MeJA (Sigma-Aldrich, USA), and then sampled at 0, 0.5, 1.5, 3, 6, 9, 12, 24 h, water with 1% concentration of DMSO as a mock treatment. The fifth leaves collected from three plants were separately pooled for each determination for RNA isolation. Real-time qPCR was carried out using the SuperReal PreMix Plus (SYBR Green) kit (Tiangen, Beijing, China) on lightcycle®96 (Roche, Mannheim, Germany). Three biological repeats were measured for each sample.

### Subcellular localization of *AaPDR3*

The full-length ORF of *AaPDR3* was cloned into *Bam*HI and *Xba*I sites of pHB-GFP vector. The recombinant plasmid was introduced into *Agrobacterium tumefaciens* strain GV3101 for *A. tumefasciens*-based *Nicotiana benthamiana* leaves transient expression (Voinnet et al., 2003). To confirm the localization of AaPDR3, we co-expressed the fusion protein GFP-AaPDR3 and the plasma membrane protein PIP1-mCherry in tobacco leaf epidermal cells. The GFP signal was observed after 2–3 days by Leica TCS SP5-II confocal laser microscopy (Leica, Wetzlar, Germany).

### Molecular cloning of *AaPDR3* promoter and promoter-GUS fusions in transgenic *A. annua*

Genomic DNA was extracted from fresh young leaves of *A. annua* using the CTAB method. The upstream region 2,059 bp of *AaPDR3* was obtained from the genome database of *A. annua*, amplified from genomic DNA with primers containing *Pst*I and *Bam*HI restriction sites and inserted into pCAMBIA1391Z vector. The resulting construct was transformed into *A. annua* plants, as described previously (Zhang et al., 2009).

### Construction of plant expression vector and transformation of *A. annua*

The 346 bp fragment of *AaPDR3* was amplified, cloned into gateway cloning vector pENTR vector using pENTR™/SD/D-TOPO® Cloning Kit (Invitrogen, Carlsbad, CA, USA), and then transferred to the destination vector pHELLSGATE12 via the LR recombination reaction (Invitrogen). The recombination plasmids (pHB-*GFP*-*AaPDR3* and pHELLSGATE12-*iAaDPR3*) were introduced into *A. tumefaciens* strain EHA105 and transformed into *A. annua* plants, as described previously (Zhang et al., 2009).

### Histochemical GUS staining and western blot analysis

The leaves were sampled from non-transgenic plants and transgenic plants for the histochemical GUS staining (Jefferson, 1987). The photographs were taken using an optical microscope (OLYMPUS, Japan). Two hundred milligrams of young leaves were powdered in liquid nitrogen, solubilized in the 2 volumes of buffer (100 mm Tris-HCl [pH 8], 50 mm KCl, 10 mm MgCl2, 20 mm DTT, and 2% Trixon-100) containing the protease inhibitors Cocktail and 1 mM phenylmethylsulfonylfluoride for 20 min on ice and centrifuged at 10,000 g for 10 min at 4°C twice. The supernatant was denatured by 2x sample buffer (125 mm TrisHCl [pH 6.8], 20% glycerol, 4% SDS, 200 mm DTT, and 0.05% bromophenol blue), incubated at 60°C for 15 min and clarified by centrifugation at 10,000 g for 1 min. The protein samples were separated on 8% SDS-PAGE gels and transferred onto nitrocellulose filters (0.45 μm pore size) (Millipore, USA). The membranes were blocked in 5% (w/v) non-fat milk powder for 2 h, and incubated with a 1:20,000 dilution of the primary antibody (Abmart, China) at 4°C overnight. The membranes were washed, incubated with a 1:10,000 dilution of goat anti-mouse alkaline phosphatase-conjugated secondary antibody (Sigma, USA), and detected using eECL Western Blot Kit (Kangwei Bio Inc., China).

### GC-MS analysis

The fresh samples were ground into fine powder in liquid nitrogen and freeze-dried for 72 h at −50°C. Fifty milligrams powder was suspended in 4 mL chromatographic-grade hexane in 10 mL glass tube with 100 μL trans-farnesol (77.6 μg/mL) as the internal standards, vigorously vortexed for 1 min and extracted for 40 min in an ultrasonic processor (JYD-650; Shanghai Zhisun Instrument Co. Ltd, China). The samples were centrifuged at 4,000 g for 10 min. The supernatants were filtered through 0.25-μm-pore-size filters, then concentrated and redissolved in 200 μL chloroform. GC-MS analysis was performed according to the methods described previously (Zhang et al., 2009). Three biological repeats were measured for each sample. Germacrene D was purchased from ChemFaces. B-caryophyllene and β-farnesene were purchased from Sigma-Aldrich.

### Quantification of artemisinin by HPLC-ELSD

The leaves of *A. annua* were collected, dried for 48 h at 50°C and ground into powder. One hundred milligrams of powder was extracted with 1 mL methanol for 30 min in an ultrasonic processor twice. The samples were centrifuged at 10,000 g for 10 min. The supernatants were filtered through 0.25-μm-pore-size filters and analyzed by the Waters Alliance 2695 HPLC system coupled with a Waters 2420 ELSD detector (Milford, USA) (Zhang et al., 2009). Three biological repeats were measured for each sample.

### Functional analysis of AaPDR3 in yeast cells

*AaPDR3* was cloned into the *Spel*I and *Pst*I sites of pDR196 by In-Fusion PCR cloning kits (Clontech, Palo Alto, CA, USA). The recombinant plasmid was transformed the strain AD1234567833 by the lithium acetate method. The yeast transformant was incubated in 50 mL SD medium (-uracil) at 29°C with shaking at 180 rpm, harvested at A600 = 1.0, and suspended by 50 mL half-strength SD medium (-uracil) containing β-caryophyllene, β-farnesene, and germacrene D, respectively. The cells were cultivated at 29°C with shaking at 180 rpm, harvested at the indicated times by centrifugation, washed twice with sterile water. The cells were disrupted with acid-washed glass beads in methanol for 15 min at 30 Hz (Yu and De Luca, 2013). Yeast cells were incubated in the culture media in the range of 0–1,200 μM β-caryophyllene for 1.5 h at pH 5.9. The cells harvested at the indicated times by centrifugation, washed twice with sterile water. Samples were centrifuged and filtered for GC-MS analysis.

## Results

### Isolation and characterization of *AaPDR3*

**Figure 1:**
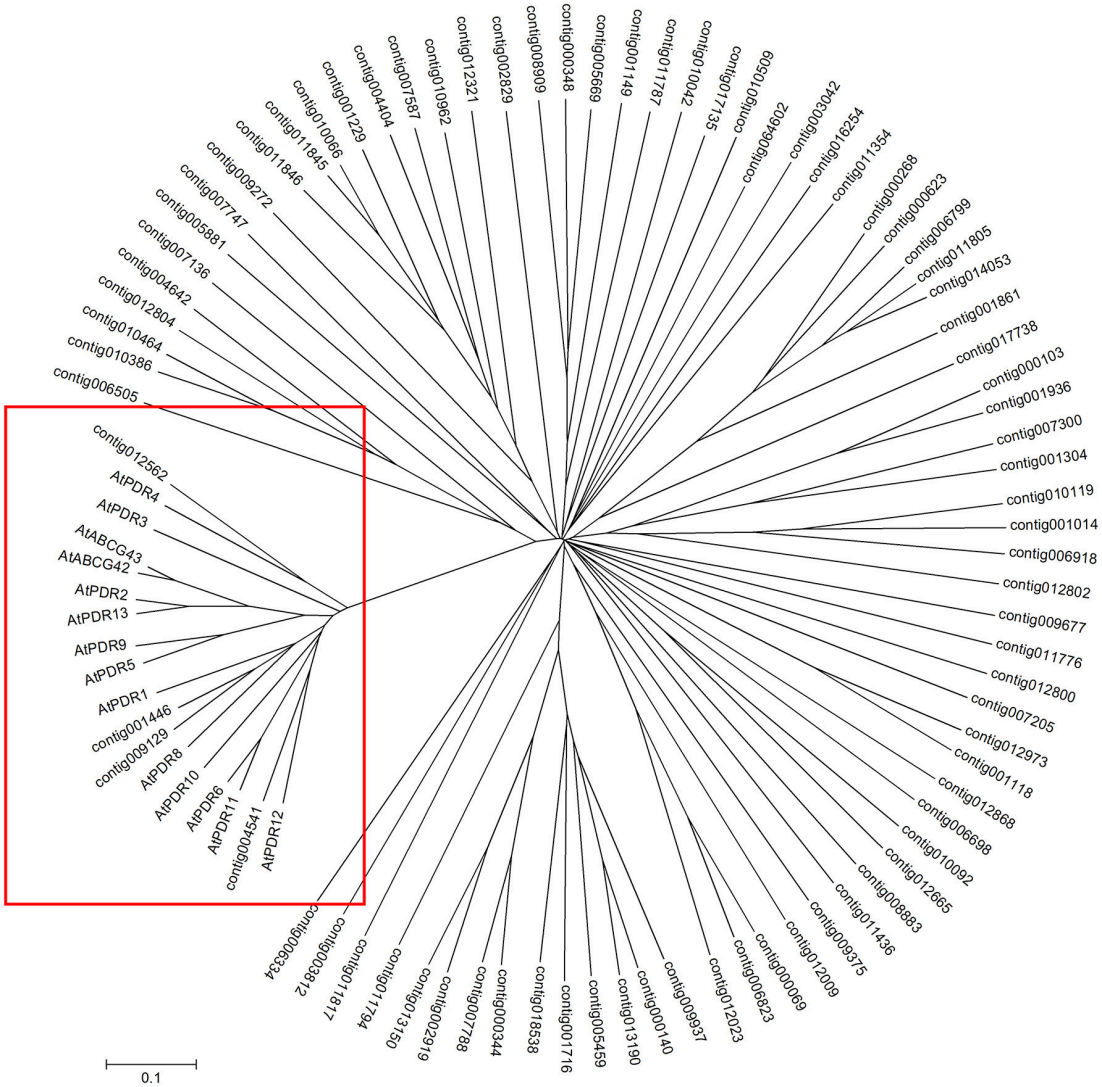
**Phylogenetic tree showing the relationship ABC transporters expressed in T-shape trichomes of ***A. annua*** compared with some PDR transporters from ***Arabidopsis*****. The tree presented here is a neighbor-joining tree based on amino acid sequence alignment.

**Figure 2:**
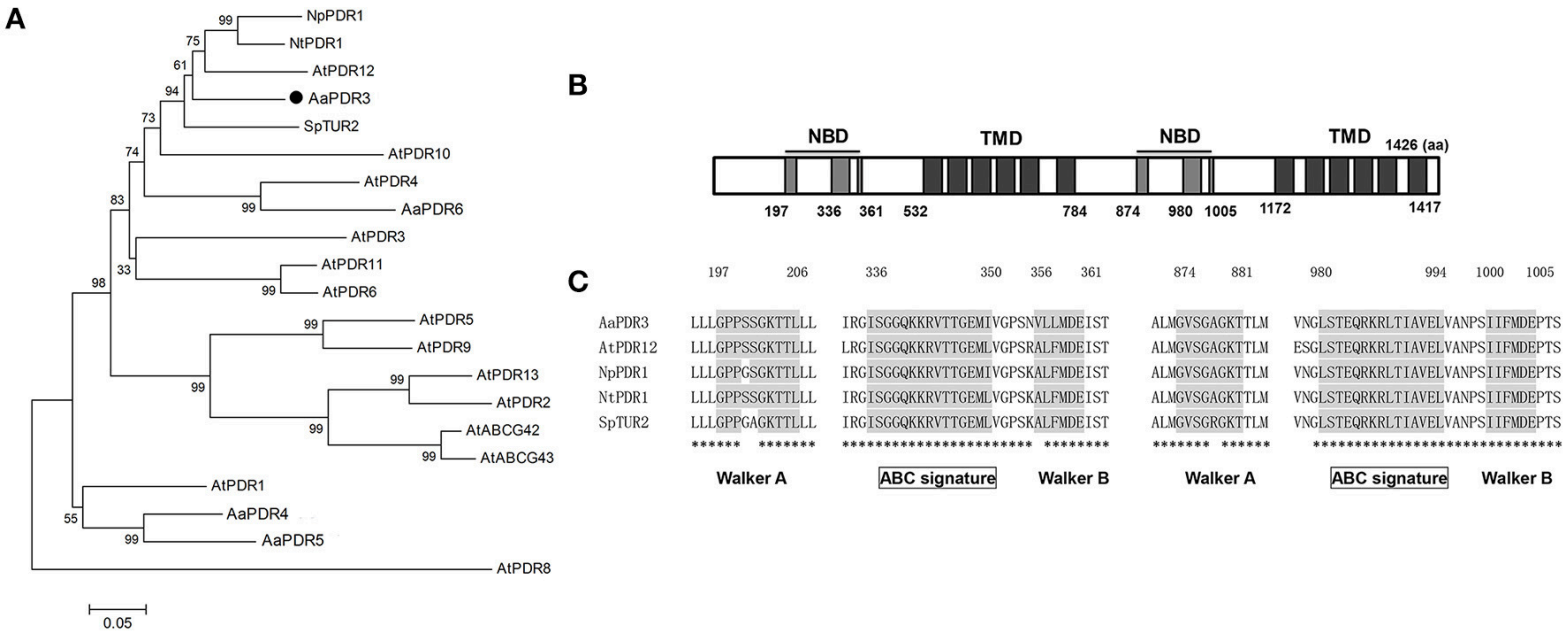
**Sequence analysis of AaPDR3. (A)** Phylogenetic analysis of PDR proteins from *A. annua* and some known PDR transporters from *Arabidopsis, N. plumbaginifolia* NpPDR1, *N. tabacum* NtPDR1, and *S. polyrrhiza* SpTUR2. The tree presented here is a neighbor-joining tree based on amino acid sequence alignment. **(B)** The structure of AaPDR3 was predicted by scanning the deduced amino acid sequence. NBD and TMD indicate the predicted location of NBDs and TMDs, respectively. **(C)** Multiple alignment of the conserved domain of known PDR transporters involved in terpene transport has the high conservation in plants. The Walker A, Walker B, and ABC signature motifs are shown with shading. The identical amino acid residues in are marked by asterisks.

Several studies have confirmed that many sesquiterpenes, with important biological functions, are produced in plant flower, leaf, secretory organ and root under constitutive, and stress conditions (Tholl, 2006). So we isolated T-shape trichomes from the capitulum of *A. annua* with laser capture microdissection and generated an RNA-Seq data based on RNA isolated from T-shape trichomes. Based on the T-shape trichomes transcriptome databases, we identified the 88 putative ABC transporters by performing a BLASTP analysis using *Arabidopsis* ABC transporter protein sequences as queries (Supplementary Information Data 1). We performed the phylogenetic analysis between PDR subfamily transporters found in *Arabidopsis thaliana* and the putative ABC transporters from *A. annua*. The result showed that four proteins were clustered with PDR transporters from *Arabidopsis thaliana*, and four PDR proteins (contig012562, contig001446, contig009129, and contig004541) were clustered with PDR transporters from *Arabidopsis* (Figure 1). Subsequently, we performed a phylogenetic tree analysis with the predicted amino acid sequences of four candidate PDR proteins and some PDR transporters containing *Arabidopsis* PDR transporters, NpPDR1, NtPDR1 and SpTUR2, showing that contig004541 protein sequence was similar to that of PDR proteins (AtPDR12, NpPDR1, NtPDR1, and SpTUR2) involved in terpene transport (Figure 2A). Therefore, this gene, named *AaPDR3*, was further examined as the candidate gene. *AaPDR3*, which is 4,278 bp in length, encodes a protein of 1,425 amino acids. This protein, belonging to the full-length size PDR subfamily, contains two nucleotide-binding domains (NBD) and two transmembrane domains (TMD; Figure 2B). Compare the conserved domain of known PDR transporters involved in terpene transport exhibited the high conservation in plants (Figure 2C). Besides, we analyzed the expression of *AaPDR3* after the treatment with 100 μM MeJA, showing that MeJA induced the expression of *AaPDR3* in *A. annua* (Figure S2).

### Expression of *AaPDR3* is tissue-specifically and developmentally regulated in *A. annua*

**Figure 3:**
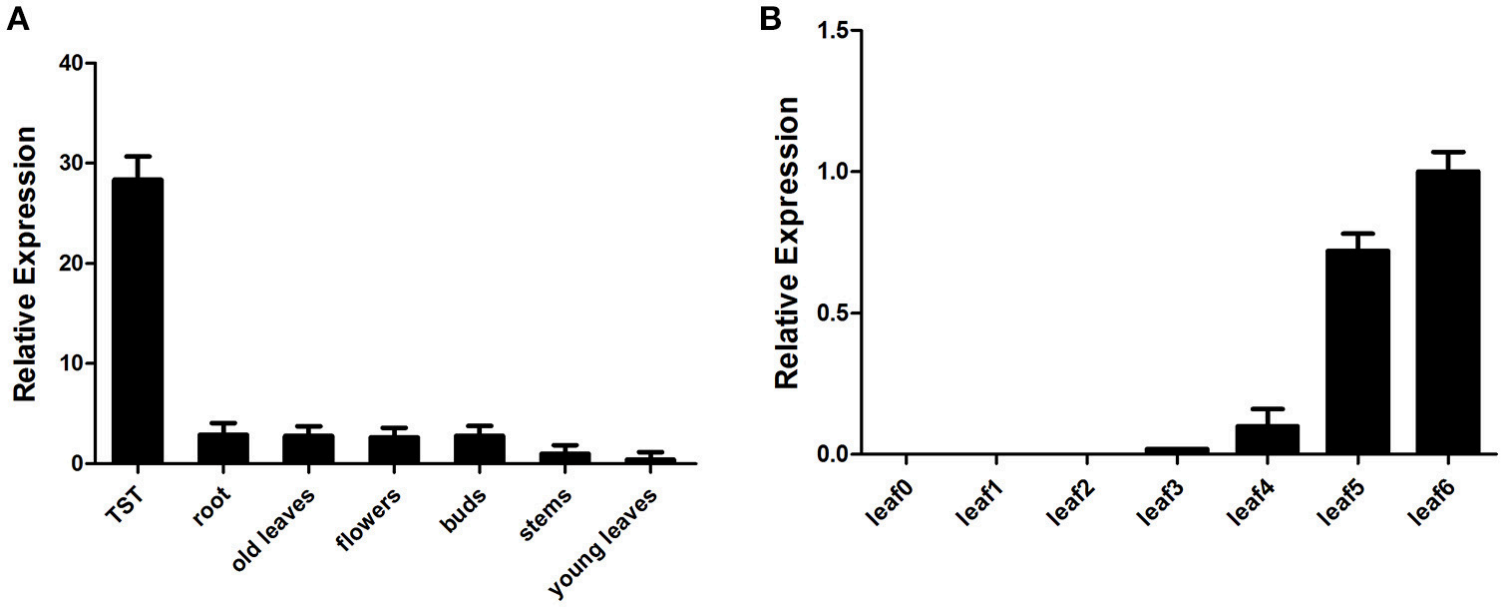
**Real-time PCR analysis for ***AaPDR3*** expression. (A,B)** Relative expression of *AaPDR3* in **(A)** TST (T-shaped trichomes), roots, stems, old leaves, young leaves, buds, flowers, and **(B)** leaves of different developmental ages of *A. annua*. *ACTIN* was used as internal control. The error bars represent the means ± *SD* (standard deviation) from three technical replicates.

Previous studies with *CPS* and *BFS* showed that the biosynthesis of related sesquiterpenes took place in roots, stems, leaves, and flower buds where they may play roles in defending the plant against fungal and worm attack (Lv et al., 2016). Consistent with these findings, investigation of *AaPDR3* transcript level by RT-qPCR revealed that *AaPDR3* expression level was the highest in T-shaped trichomes (Figure 3A). *AaPDR3* is also determined in roots, stems, leaves, and flower buds (Figure 3A). Moreover, we analyzed the expression of *AaPDR3* in leaves at different developmental stages. The expression level is the lowest in the youngest leaf (leaf0) and increased gradually with the leaves aging (Figure 3B).

### AaPDR3 is located to the plasma membrane

**Figure 4:**
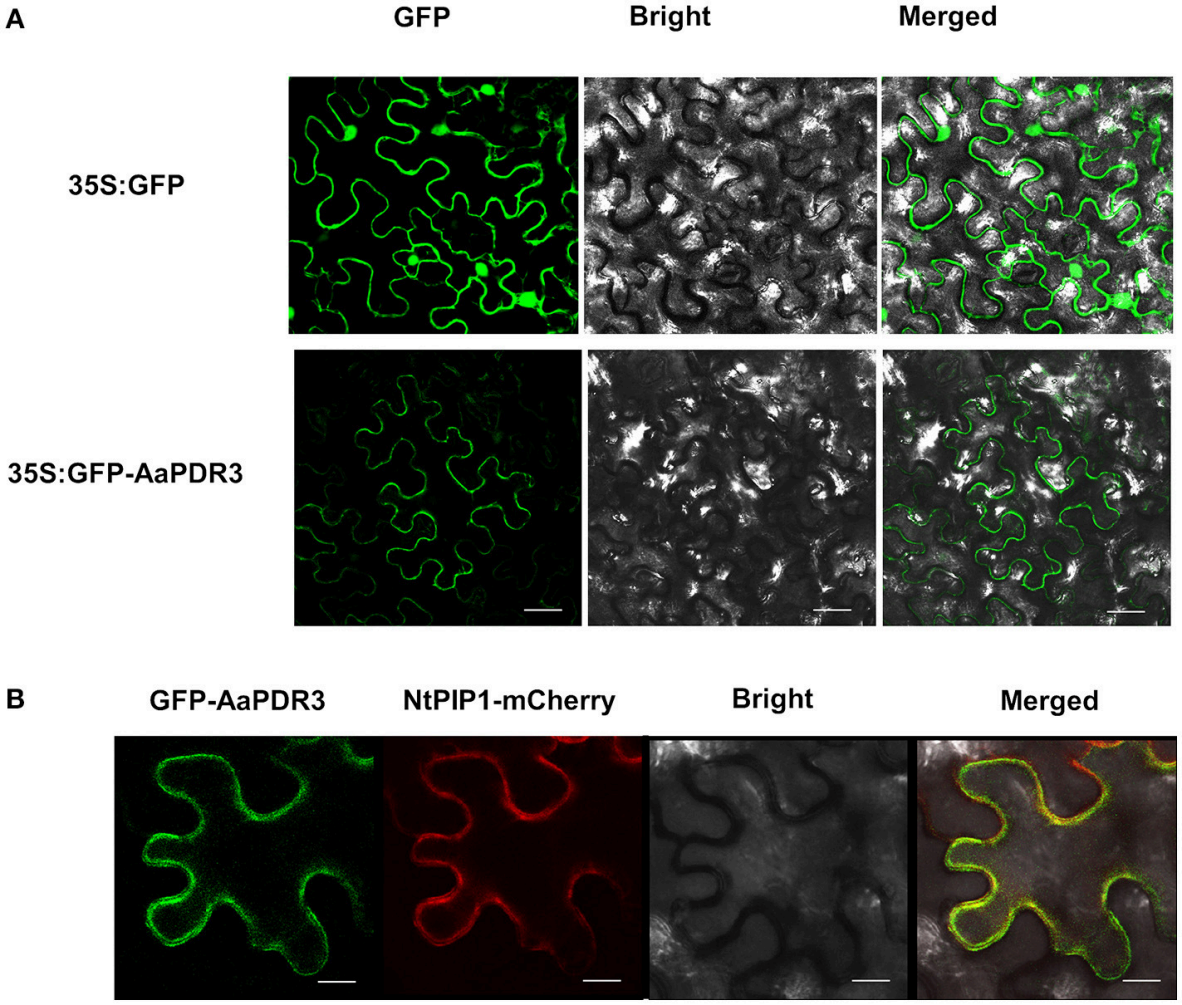
**The subcellular localization of ***AaPDR3***. (A)** Localization of AaPDR3 in tobacco leaves. Bars = 10 μm. **(B)** AaPDR3 protein co-localized with plasma membrane integral protein PIP1 on the plasma membrane of tobacco leaves determined through confocal microscopy. Bars = 40 μm.

Analysis of the encoded AaPDR3 protein by the subcellular prediction programs (Predotar: https://urgi.versailles.inra.fr/predotar/predotar.html; WoLF PSORT: http://www.genscript.com/psort/wolf_psort.html) predicted that this protein has no N-terminal signal peptide and is located to the plasma membrane. To examine the subcellular localization of AaPDR3 protein, the green fluorescent protein (GFP) fused to the N-terminal domain of *AaPDR3* under CaMV35S promoter was transiently expressed in tobacco leaves. Results showed that GFP fluorescence of leaves expressing *GFP*-*AaPDR3* was only observed in the plasma membrane (Figure 4A). The GFP fused to the N-terminal domain of AaPDR3 together with the established plasma membrane marker PIP1 (Siefritz et al., 2002) fused to mCherry were transiently co-expressed in tobacco leaves. The GFP-AaPDR3 green fluorescent signal was colocalized to the plasma membrane with PIP1-mCherry (Figure 4B). The results were consistent with those from prediction programs, indicating that AaPDR3 was localized in the plasma membrane and might function as a transporter.

### *AaPDR3* is expressed in T-shaped trichomes and roots of *A. annua*

**Figure 5:**
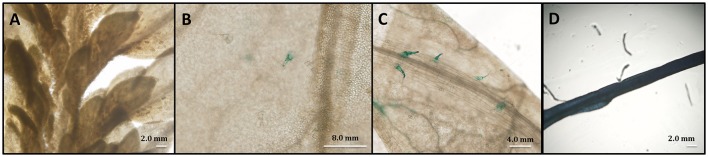
*****AaPDR3*** is mainly expressed in T-shape trichomes and roots**. The expression of the *proAaPDR3*-GUS was observed in **(A)** the first leaf, **(B)** the fifth leaf, **(C)** the sixth leaf, and **(D)** the root.

To further investigate the tissue-specific expression pattern of *AaPDR3* in *A. annua*, a 2,059-bp genomic fragment corresponding to the predicted *AaPDR3* promoter sequence in our genome database was cloned from *A. annua* and then fused to *GUS* reporter gene. The recombinant plasmid was introduced into *A. annua* plants. GUS activity was analyzed in different tissues in *A. annua*. The result showed that GUS-staining was primarily restricted to T-shaped trichomes of old leaves in transgenic plants (Figures 5A–C). And GUS activity was also observed in roots in transgenic plants (Figure 5D), where a large number of sesquiterpenes are synthesized and stored.

### AaPDR3 affects sesquiterpenes β-caryophyllene biosynthesis in *A. annua*

**Figure 6:**
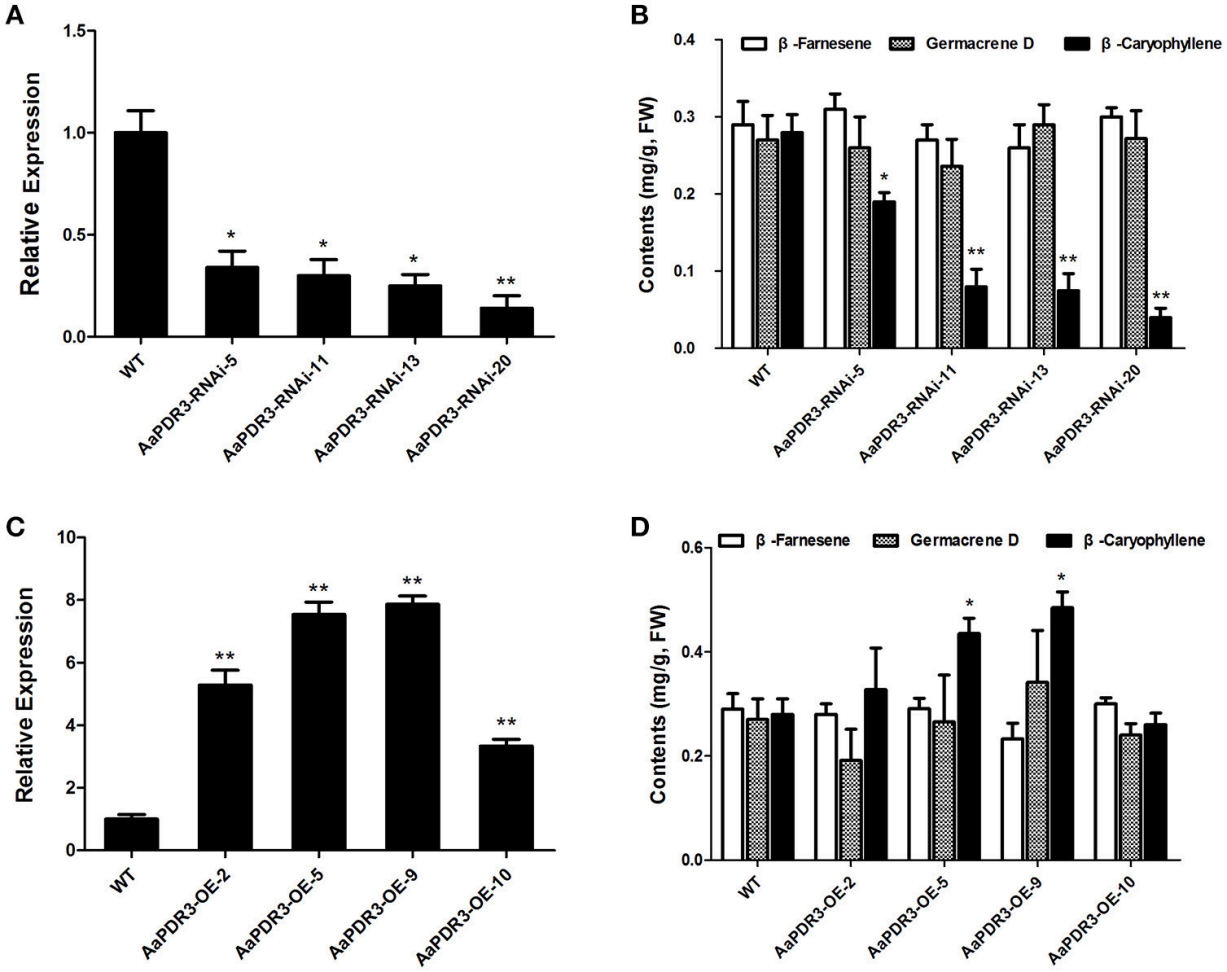
**Comparative analyses of ***AaPDR3*** gene expression and sesquiterpene analyses in wild type (WT), in four ***AaPDR3***-RNAi and four ***AaPDR3***-overexpression plants. (A)** Relative expression of *AaPDR3* in *AaPDR3*-RNAi transgenic *A. annua* lines. **(B)** The contents of β-farnesene, β-caryophyllene, and germacrene D in *AaPDR3*-RNAi transgenic *A. annua* lines. **(C)** Relative expression of *AaPDR3* in *AaPDR3*-overexpression transgenic *A. annua* lines. **(D)** The contents of β-farnesene, β-caryophyllene, and germacrene D in *AaPDR3*-overexpression transgenic *A. annua* lines. The error bars represent the means ± *SD* from three biological replicates, and asterisks indicate statistically significant differences compared with WT. ^*^P < 0.05, ^**^P < 0.01.

To explore the function of AaPDR3 in *A. annua*, we generated 34 *AaPDR3*-RNAi transgenic plants using an RNAi strategy under the control of the CaMV35S promoter. In the RNAi transgenic plants, four independent lines with 14–34% observably downregulated *AaPDR3* expression (Figure 6A) were selected for the detailed metabolic profiling analysis by gas chromatography-mass spectrometry (GC-MS) analysis (Figure S3). In contrast with the wild type, the suppression of *AaPDR3* led to a 32–86% reduction of β-caryophyllene content (Figure 6B), while germacrene D and β-farnesene levels remained unchanged in *AaPDR3*-*RNAi* lines compared with wild type (Figure 6B). These data indicate that the repression of *AaPDR3* markedly results in the suppression of sesquiterpene β-caryophyllene biosynthesis in *A. annua*.

*AaPDR3* under the control of CaMV35S promoter was overexpressed in *A. annua*. We obtained 28 *AaPDR3*-overexpressing transgenic plants. Investigation of *AaPDR3* transcript levels by qRT-PCR showed that the *AaPDR3* expression was significantly increased in four *AaPDR3*-overexpression lines (Figure 6C). The four independent transgenic lines were identified by Western blot (Figure S4). Consistent with a role involved in sesquiterpenes biosynthesis transport *in planta*, the level of β-caryophyllene was increased to 0.48 mg/g FW in *AaPDR3*-overexpression lines compared to control (0.28 mg/g FW; Figure 6D). Little increases of β-farnesene and germacrene D were observed in *AaPDR3*-overexpression plants compared to wild type (Figure 6D). Taken together, AaPDR3 is involved in the sesquiterpene β-caryophyllene biosynthesis in *A. annua*. Moreover, the repression of *AaPDR3* observably increased artemisinin contents in the RNAi plants (Figure S5).

### AaPDR3 functions as β-caryophyllene transporter in yeast strain AD1-8

**Figure 7:**
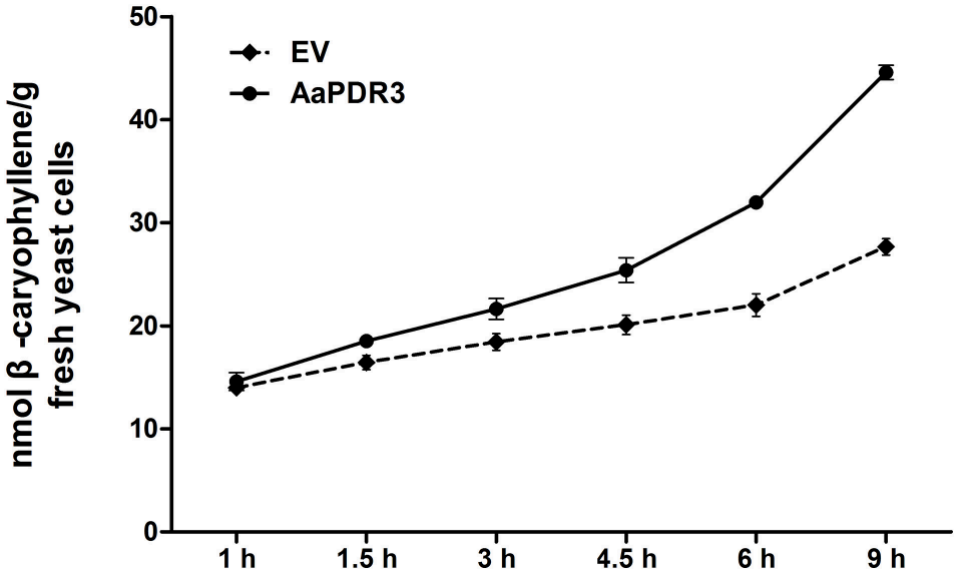
**Time-dependent uptake of β-caryophyllene by AD1-8 yeast cells expressing ***AaPDR3*** and transformed with the empty vector (EV)**. Yeast was incubated in half-strength SD medium containing 100 μM β-caryophyllene at pH 5.9. The error bars represent the means ± *SD* from three biological replicates.

A heterologous yeast expression system is an informative approach for elucidating the function of transporters (Morita et al., 2009; Shitan et al., 2013; Yu and De Luca, 2013). To investigate the function of AaPDR3 transporter, we expressed the *AaPDR3* cDNA in the yeast strain AD12345678 lacking eight major ABC transporters and one transcription factor (Decottignies et al., 1998). Then we selected β-caryophyllene, β-farnesene, and germacrene D as the candidate substrates, respectively. The yeast cells of *AaPDR3* transformant and the control (transformed with the empty vector PDR196) were incubated in half-strength Synthetic Dextrose (SD) medium contained 100 μM of each substrates, and the intracellular contents were quantitatively analyzed by LC-MS. Yeast cells expressing *AaPDR3* accumulated more β-caryophyllene than the control along the same time course (Figure 7). The *AaPDR3* transformants accumulated >44 nmol of β-caryophyllene per gram of cells compared with the control cells the contained almost 27 nmol at 9 h treated by 100 μM β-caryophyllene (Figure 7). The result demonstrated that expression of AaPDR3 increased β-caryophyllene influx. Both *AaPDR3* transformants and the control were incubated in the culture media in the range of 0–1,200 μM β-caryophyllene. β-caryophyllene uptake by AaPDR3 followed Michaelis-Menten kinetics with K_m_ of 63.47 ± 8.81 pmol β-caryophyllene and a maximum transport rate *V*_max_ of 80.89 ± 2.46 pmol/g fresh yeast cells/min (Figure S6). No significant differences in the β-farnesene contents accumulated in *AaPDR3* expressing yeast cells compared to that in the control group, as well as germacrene D (Figure S7). These results indicated that AaPDR3 was highly specific for the β-caryophyllene transport compared with β-farnesene and germacrene D in yeast.

## Discussion

### AaPDR3 mediates the β-caryophyllene biosynthesis in *A. annua*

The terpenoids are one of the largest groups of plant secondary metabolites (Croteau et al., 2000). Interestingly, the terpenoids are often transported from the cells where these compounds or metabolic intermediates are synthesized to neighboring cells, or even to other tissues or remote organs to be stored. Therefore, many transporter proteins participate in this biology process (Yazaki, 2005). Recently, the ATP-binding cassette (ABC) transporters have been reported to largely contribute to membrane transport of terpenoids in plants, especially for PDR subfamily (Jasiński et al., 2001; Van Den Brûle et al., 2002; Crouzet et al., 2013). Most of identified PDR transporters were expressed in specific tissues. For example, *AtPDR8* is predominately expressed in roots and leaves. *AtPDR2, AtPDR6, AtPDR9*, and *AtPDR13* are exclusively expressed in roots but not in shoots in *Arabidopsis*, while *AtPDR14* is expressed only in shoots (Den Brule and Smart, 2002). The expression of *AtPDR5* is mainly detected in roots and stems (Bienert et al., 2012). *NtABCG5*/*PDR5*, from *N. tabacum*, was highly expressed in the petals, stem and roots (Bienert et al., 2012). NtPDR1 was detected in stem and leaf tissues (Crouzet et al., 2013). These findings show that *PDR* genes are predominately expressed in roots and leaves. In plants, roots and leaves are the important tissues connected with environment. In this study, we characterized a PDR transporter AaPDR3 in *A. annua*. In our investigation, AaPDR3 was mainly active in old leaves, flowers, buds, and roots (Figure 3A). Notably, the GUS analysis exhibited that AaPDR3 was specifically expressed in T-shaped trichomes of old leaves and roots (Figure 5C). Likewise, AaPDR3 exhibited the tissues-specific expression pattern, suggesting that AaPDR3 plays an important role in the defensive compounds biosynthesis in T-shaped trichomes, flowers, buds, and roots. Moreover, the transcript level of *CPS* was also detected in leaves, flowers, buds, and roots (Lv et al., 2016), which is in accord with that of *AaPDR3* in *A. annua*. Besides, the expression level of *AaPDR3* was barely detected in the youngest leaf (leaf0), and increased gradually with the leaves aging (Figure 3B). Although the expression of *CPS* was highest in youngest leaf (leaf0), the *CPS* transcript level was also detected with the leaves aging (Lv et al., 2016). It means that β-caryophyllene is synthesized in young leaves and old leaves. From these results, we propose that AaPDR3 as a plasma membrane β-caryophyllene cellular uptake for gathering β-caryophyllene. Then the gathered β-caryophyllene is stored in the some cells of T-shape trichomes to reduce the cell damaged.

To identify the function of AaPDR3 in *A. annua*, we used RNAi to knock down the expression of *AaPDR3*. The repression of *AaPDR3* resulted in an 86% reduction of β-caryophyllene content in *AaPDR3*-RNAi-20 transgenic *A. annua* line (Figure 6B), suggesting that AaPDR3 is essential for β-caryophyllene biosynthesis in *A. annua*. Transporters are the integral parts in metabolic networks, because they mediate multiple metabolic pathways. We speculated that the *AaPDR3* repression would result in β-caryophyllene accumulated in the cells of T-shape trichomes in *A. annua*, which would prevent the β-caryophyllene biosynthesis. Our results, together with previous findings, indicated that AaPDR3 is involved in β-caryophyllene transport and plays an indispensable role in β-caryophyllene biosynthesis. AaPDR3 transporter reported here is the first transporter related to sesquiterpenes in *A. annua*, even in family Asteraceae.

### AaPDR3 was involved in β-caryophyllene transport in yeast

Plant ABC transporters is a large and diverse group of proteins involved in the pathogen response, lipid deposition, and the transport of the phytohormones (Kretzschmar et al., 2011). Therefore, ABC transporters play an important part in plant growth, nutrition, development, and the interaction with the environment (Bird et al., 2007; Kuromori et al., 2010; Ding et al., 2011). Our results preferentially suggest that AaPDR3 is likely to be involved in the sesquiterpene β-caryophyllene transport based on four findings: (i) like other sesquiterpene transporters; the amino acid sequence of AaPDR3 is similar to that of PDR transporters involved in terpene transport (Figure 2A), (ii) the plasma membrane protein AaPDR3 is expressed in the tissues, including the T-shaped trichomes, buds, flowers, and roots, where the sesquiterpenes are synthesized (Figure 3A), (iii) increase and decrease in the *AaPDR3* transcript level influence the sesquiterpene β-caryophyllene biosynthesis (Figure 6), and (iv) when *AaPDR3* was expressed in yeast mutant AD1-8, yeast expressing *AaPDR3* only took up β-caryophyllene faster than controls containing the empty vector (Figure 7). In fact, some ABC transporters are reported to have broad substrate specificity (Kolaczkowski et al., 1998). For example, PDR5 transporter from yeast was confirmed to export some compounds which had different structure and function (Wolfger et al., 2001; Lamping et al., 2010). In *Arabidopsis*, AtPDR12 is an ABA-uptake transporter in the guard cells and other cells (Kang et al., 2010). The plasma membrane transporter, AtPDR12, also contributes to the resistance of lead (Lee et al., 2005). When the yeast cells expressing *AaPDR3* was incubated in SD medium contained 100 μM β-caryophyllene, β-farnesene, and germacrene D, respectively, our results showed that AaPDR3 exhibited narrow substrate specificity (Figure 7 and Figure S6).

### AaPDR3 affects the artemisinin biosynthesis in *A. annua*

Amazingly, we found that knockdown of *AaPDR3* resulted in an increase in artemisinin content in *AaPDR3*-RNAi transgenic plants (Figure S5). *AaPDR3* is a specific-expressed transporter gene in T-shape trichomes (Figure 4C), whereas artemisinin is specially synthesized in glandular trichomes. As we known, blocking the competitive pathways of artemisinin biosynthesis is very useful to improve the artemisinin content (Zhang et al., 2009; Lv et al., 2016). Both the artemisinin and dihydroartemisinic acid contents were increased, when *CPS* was suppressed by anti-sense in *A. annua* (Lv et al., 2016). The β-caryophyllene content in *A. annua* was up to 5–10% of the total essential oil (Brown, 2010). When *AaPDR3* was down-regulated by RNAi in *A. annua*, the β-caryophyllene content was observably reduced in transgenic plants (Figure 6B), which might lead to the carbon altered through FPP to artemisinin biosynthetic pathway.

## Accession numbers

*AaPDR3* (KR153482), *AtPDR1* (NM_112505.4), *AtPDR2* (NM_117611.5), *AtPDR3* (NM_128548.4), *AtPDR4* (NM_128248.2), *AtPDR5* (NM_001336647.1), *AtPDR6* (NM_129195.6), *AtPDR7* (NM_101389.3), *AtPDR8* (GQ374243.1), *AtPDR9* (NM_115208.4), *AtPDR10* (NM_001339062.1), *AtPDR11* (NM_105366.4), *AtPDR12* (NM_001332173.1), *AtPDR13* (NM_001341001.1), *AtABCG42* (NM_001203808.2), *AtABCG43* (NM_148328.3), NpPDR1 (Q949G3.1), NtPDR1 (Q76CU2.1), SpTUR2 (O24367.1).

## Author contributions

XF and KT designed the research and drafted the manuscript. XF and PS performed the experiments. XF, QH, QS, YM, and PL carried out vector construct, expression analysis, transgene plant generation, subcellular localization and yeast assay. YT, QP, TY, MC, XH, LL, YW, and XS revised the manuscript. All authors approved the manuscript.

## Funding

This work was supported by the China National Transgenic Plant Research and Commercialization Project (Grant No. 2016ZX08002-001), China National High Technology Research and Development Program (Grant No. 2011AA100605), and Shanghai Jiao Tong University Agri-Engineering Program (Grant No. AF1500028).

### Conflict of interest statement

The authors declare that the research was conducted in the absence of any commercial or financial relationships that could be construed as a potential conflict of interest.

## Supplementary material

Figure S1 **The sesquiterpene biosynthesis in ***Artemisia annua*****. HMGR, 3-hydroxy-3- methylglutaryl coenzyme A reductase; DXS, 1-deoxy-D-xylulose-5-phosphate synthase; DXR, 1-deoxy-D-xylulose 5-phosphate reductase. FPS, farnesyl diphosphate; ADS, amorpha-4,11-diene synthase; CPR, cytochrome P450 reductase; CYP71AV1, cytochrome P450 monooxygenase; DBR2, artemisinic aldehyde D-11(13)-double bond reductase; ALDH1, aldehyde dehydrogenase 1; CYB5 and ADH1, cytochrome b5 monooxygenase and alcohol dehydrogenase. CPS, beta-caryophyllene synthase; BFS, beta-farnesene synthase; GAS, germacrene A synthase; ECS, 8-epicedrol synthase.

Figure S2 **The expression of ***AaPDR3*** was induced by MeJA treatment**. *ACTIN* was used as internal control. The error bars represent the means ± *SD* from three biological replicates.

Figure S3 **Total ion chromatographs of metabolites from leaves of ***A. annua*****.

Figure S4 **Immunoblotting analysis of GFP-AaPDR3 protein levels in ***AaPDR3***-overexpression transgenic ***A. annua*** lines**.

Figure S5 **The content of artemisinin in overexpression and RNAi transgenic ***A. annua*** plants, respectively**. The error bars represent the means ± SD from three biological replicates, and asterisks indicate statistically significant differences compared with WT. ^**^*P* < 0.01.

Figure S6 **β-caryophyllene uptake analyses by AD1-8 yeast cells expressing ***AaPDR3*** and transformed with the empty vector (EV)**. Yeast cells were incubated in the culture media in the range of 0–1,200 μM β-caryophyllene at pH 5.9. β-caryophyllene uptake by AaPDR3 followed Michaelis- Menten kinetics with K_m_ of 63.47 ± 8.81 pmol β-caryophyllene and a maximum transport rate *V*_max_ of 80.89 ± 2.46 pmol/g fresh yeast cells/min (*R*^2^ = 0.98). The error bars represent the means ± *SD* from three biological replicates.

Figure S7 **Time-dependent uptake of (A)** β-farnesene and **(B)** germacrene D by AD1-8 yeast cells expressing *AaPDR3* and transformed with the empty vector (EV). Yeast was incubated in half-strength SD medium containing 100 μm β-farnesene and germacrene D at pH 5.9, respectively. The error bars represent the means ± *SD* from three biological replicates.

Table S1 **Primers used in this study**.

Supplementary Information Data 1 **Four selected PDR genes in transcriptome expression in glandular and T-shaped trichomes**.
