# Supplementary figures and images for: AaPDR3, a PDR Transporter 3, Is Involved in Sesquiterpene β-Caryophyllene Transport in Artemisia annua
Source: Front Plant Sci. 2017 May 8;8:723. doi: 10.3389/fpls.2017.00723 (PMC5420590; doi:10.3389/fpls.2017.00723)

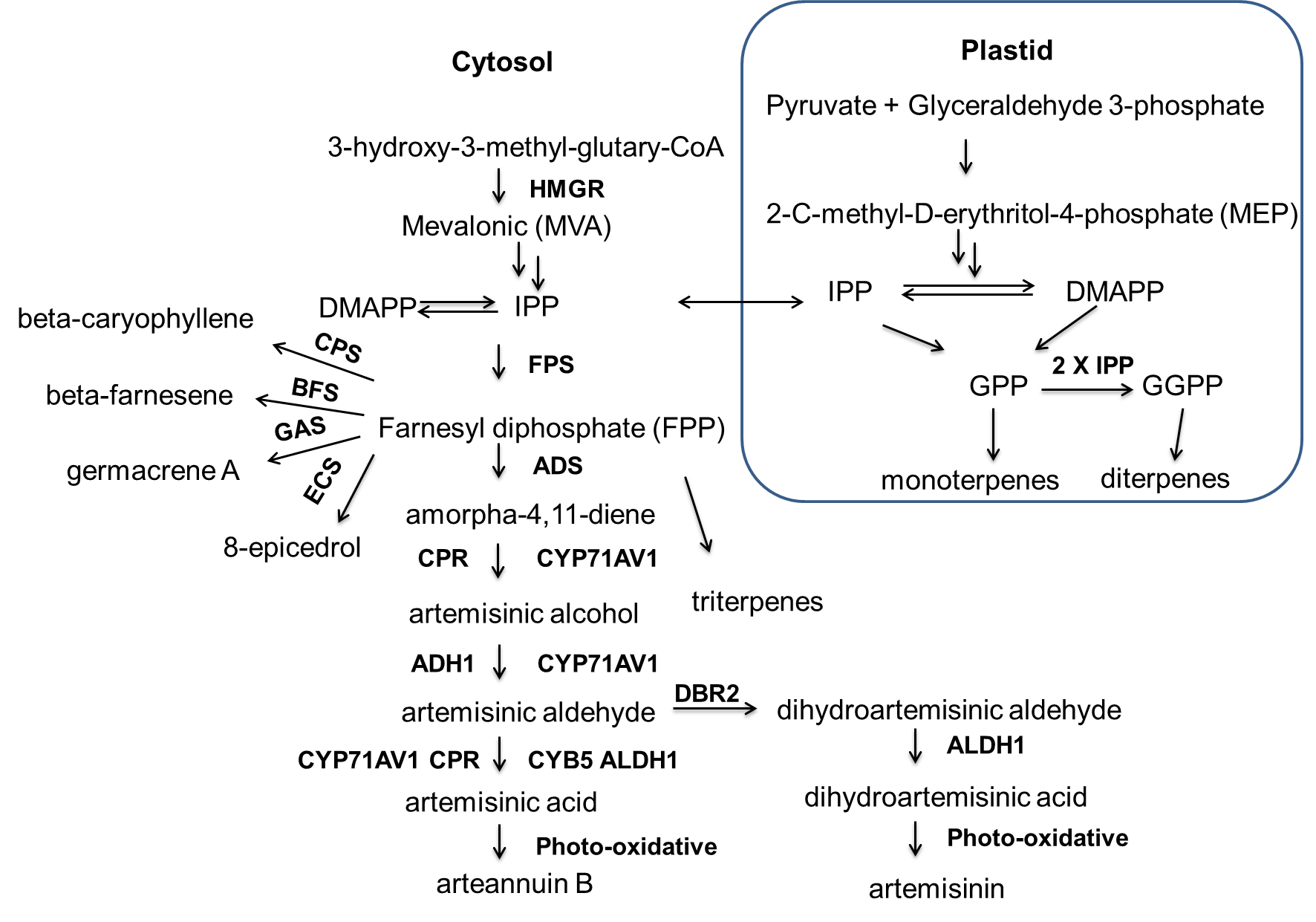

Supplement: Figure S1 — The sesquiterpene biosynthesis in Artemisia annua. HMGR, 3-hydroxy-3- methylglutaryl coenzyme A reductase; DXS, 1-deoxy-D-xylulose-5-phosphate synthase; DXR, 1-deoxy-D-xylulose 5-phosphate reductase. FPS, farnesyl diphosphate; ADS, amorpha-4,11-diene synthase; CPR, cytochrome P450 reductase; CYP71AV1, cytochrome P450 monooxygenase; DBR2, artemisinic aldehyde D-11(13)-double bond reductase; ALDH1, aldehyde dehydrogenase 1; CYB5 and ADH1, cytochrome b5 monooxygenase and alcohol dehydrogenase. CPS, beta-caryophyllene synthase; BFS, beta-farnesene synthase; GAS, germacrene A synthase; ECS, 8-epicedrol synthase. [file Image1.TIF]

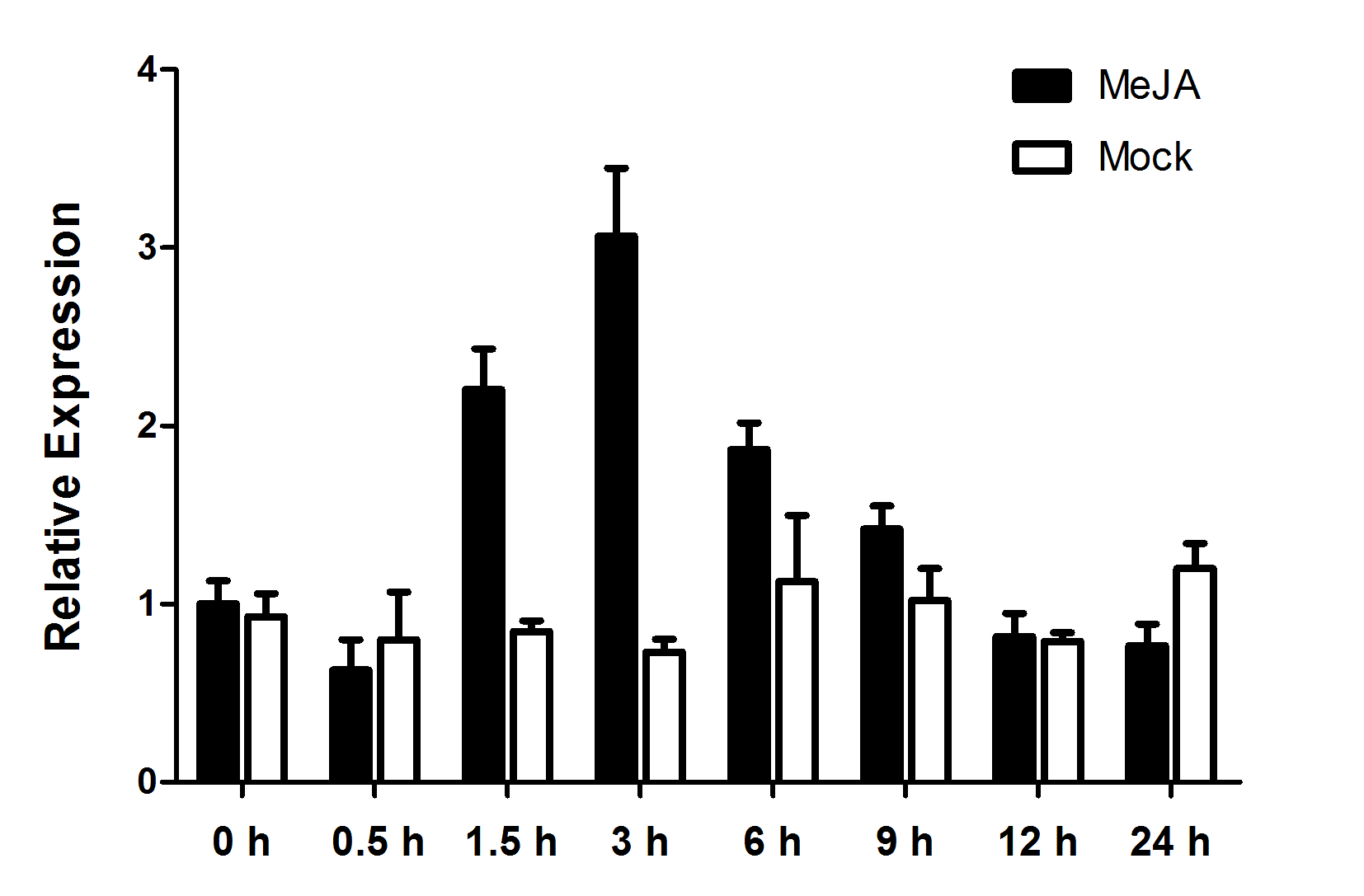

Supplement: Figure S2 — The expression of AaPDR3 was induced by MeJA treatment. ACTIN was used as internal control. The error bars represent the means ± SD from three biological replicates. [file Image2.TIF]

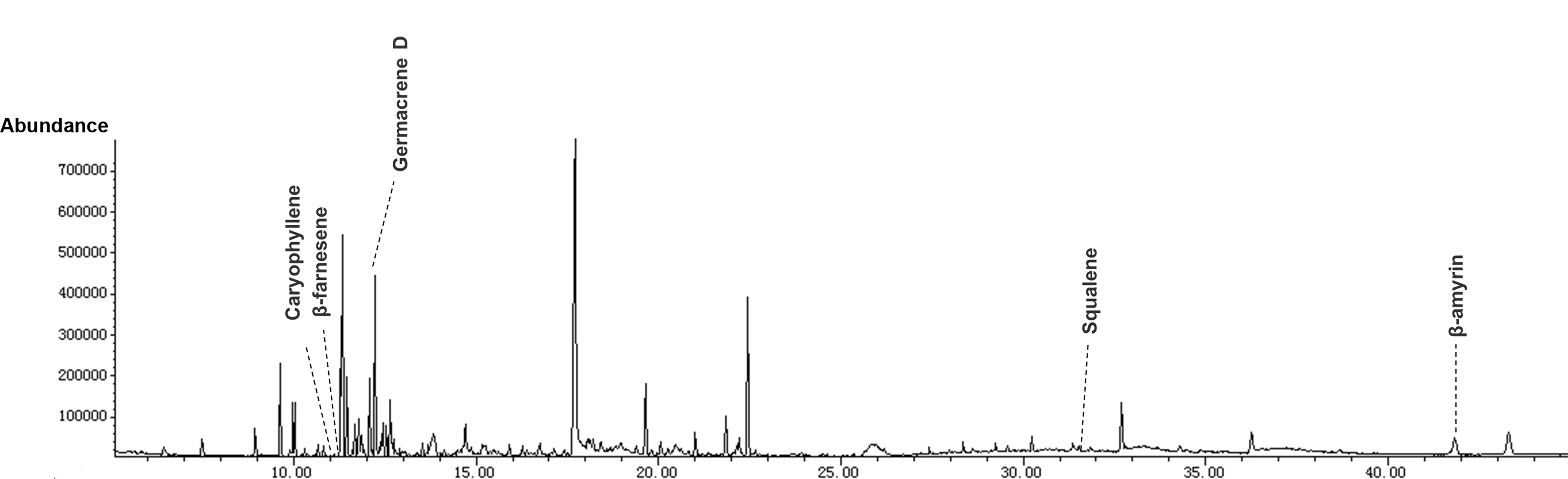

Supplement: Figure S3 — Total ion chromatographs of metabolites from leaves of A. annua. [file Image3.TIF]

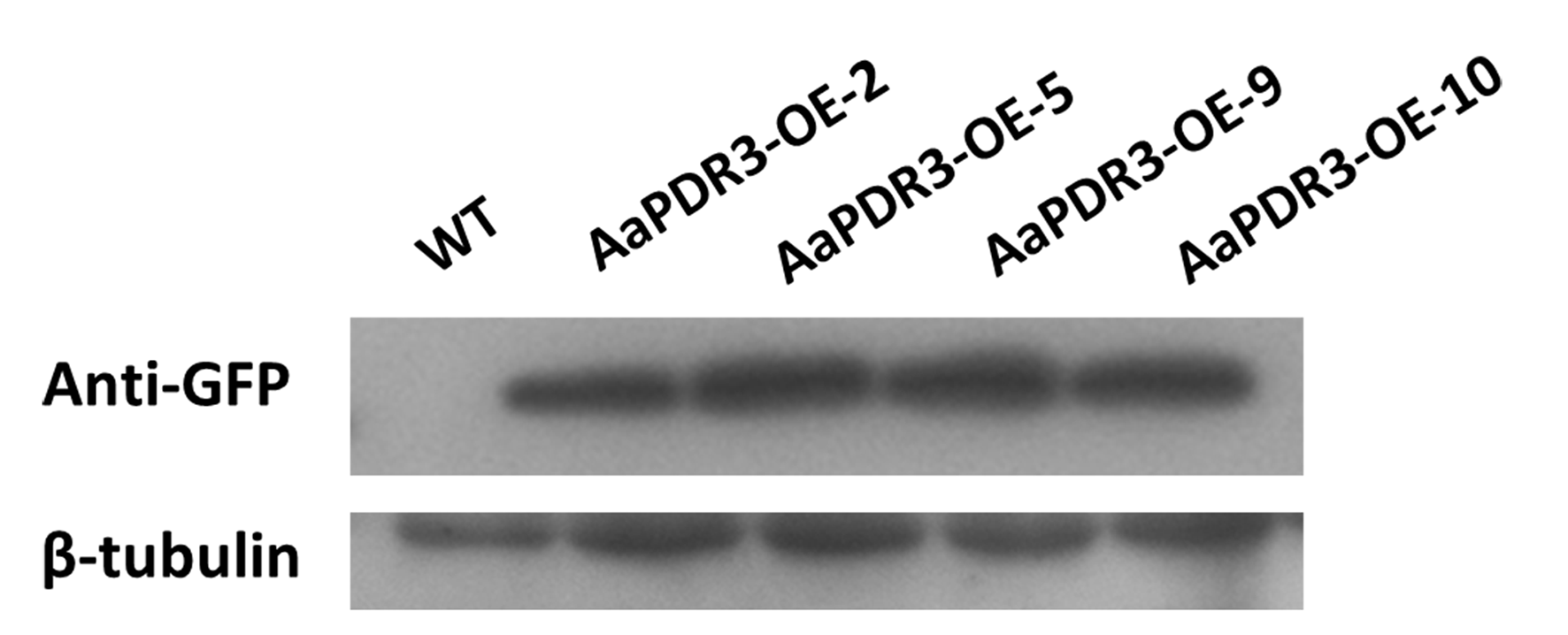

Supplement: Figure S4 — Immunoblotting analysis of GFP-AaPDR3 protein levels in AaPDR3-overexpression transgenic A. annua lines. [file Image4.TIF]

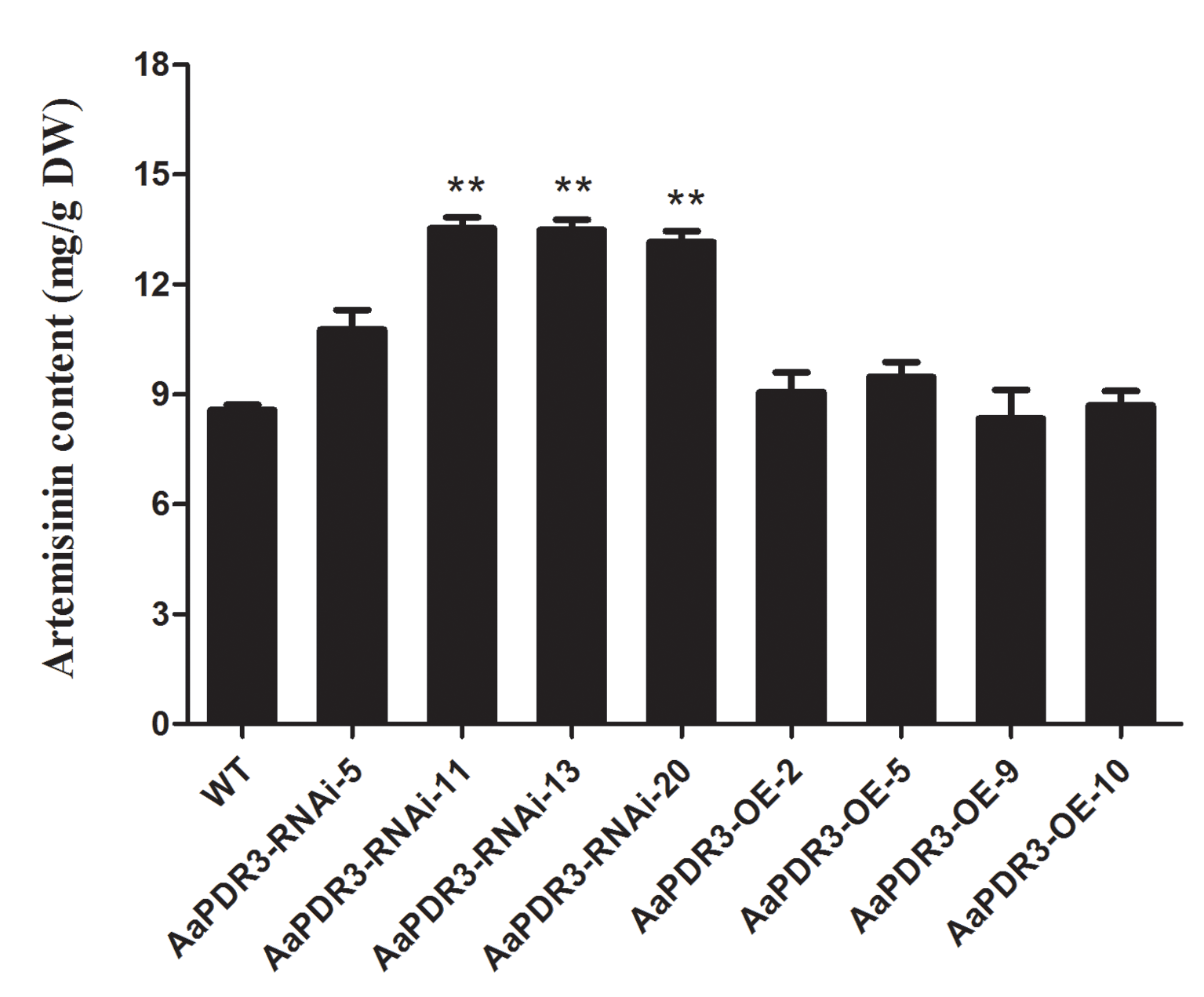

Supplement: Figure S5 — The content of artemisinin in overexpression and RNAi transgenic A. annua plants, respectively. The error bars represent the means ± SD from three biological replicates, and asterisks indicate statistically significant differences compared with WT. **P < 0.01. [file Image5.TIF]

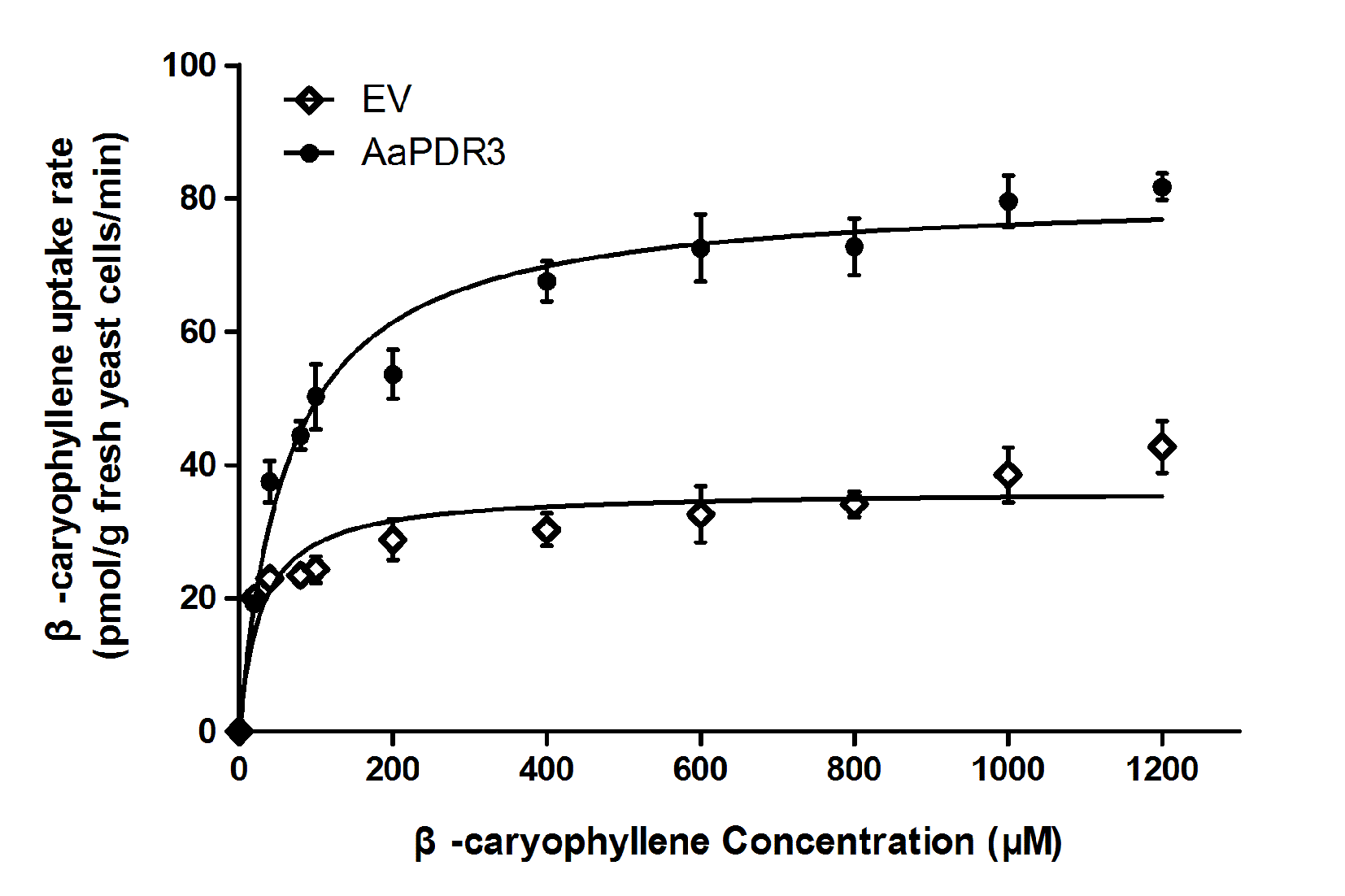

Supplement: Figure S6 — β-caryophyllene uptake analyses by AD1-8 yeast cells expressing AaPDR3 and transformed with the empty vector (EV). Yeast cells were incubated in the culture media in the range of 0–1,200 μM β-caryophyllene at pH 5.9. β-caryophyllene uptake by AaPDR3 followed Michaelis- Menten kinetics with Km of 63.47 ± 8.81 pmol β-caryophyllene and a maximum transport rate Vmax of 80.89 ± 2.46 pmol/g fresh yeast cells/min (R2 = 0.98). The error bars represent the means ± SD from three biological replicates. [file Image6.TIF]

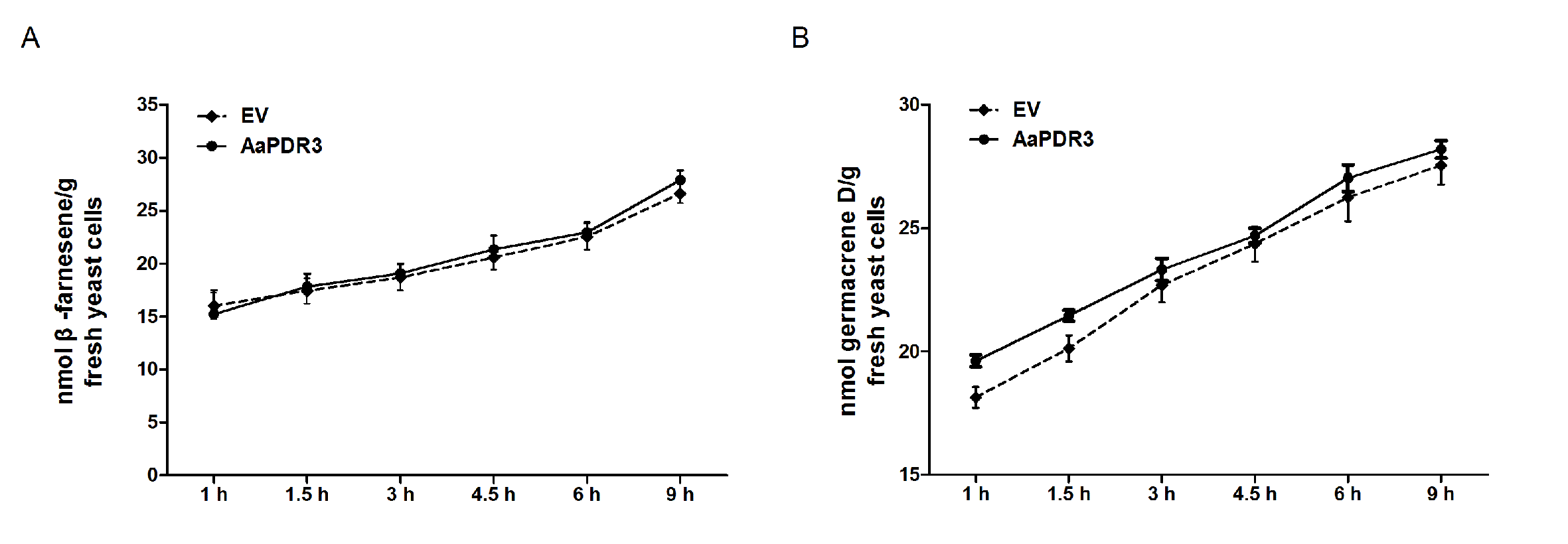

Supplement: Figure S7 — Time-dependent uptake of (A) β-farnesene and (B) germacrene D by AD1-8 yeast cells expressing AaPDR3 and transformed with the empty vector (EV). Yeast was incubated in half-strength SD medium containing 100 μm β-farnesene and germacrene D at pH 5.9, respectively. The error bars represent the means ± SD from three biological replicates. [file Image7.TIF]
