# Supplementary material for: AaPDR3, a PDR Transporter 3, Is Involved in Sesquiterpene β-Caryophyllene Transport in Artemisia annua
Source: Front Plant Sci. 2017 May 8;8:723. doi: 10.3389/fpls.2017.00723 (PMC5420590; doi:10.3389/fpls.2017.00723)
Supplement: Table S1 — Primers used in this study. [file Table1.DOCX]

**Table S1.** Primers used in this study**.**

| No. | Primer name | Use | Primer sequence 5´-3´ | Restriction enzyme |
| --- | --- | --- | --- | --- |
| 1 | AaPDR3-FP1 | cloning | TTCTCGTAGGGCTTTTTGAGCATTA | - |
| 2 | AaPDR3-RP1 | cloning | GCCATACAAAGACGCTAATAGAACTCA | - |
| 3 | BamHI-AaPDR3-FP | cloning | CGGGATCCATGGATGGAAGTGATATTTATA | *BamH*I |
| 4 | AaPDR3-XbaI-RP | cloning | GCTCTAGACTATCTCTTTTGGAAATTAA | *Xba*I |
| 5 | AaPDR3-RNAi-FP | cloning | CACCATGGATGGAAGTGATATTTATAA | - |
| 6 | AaPDR3-RNAi-RP | cloning | CATCAACTTCTTCTGAAGGTCCAG | - |
| 7 | Pro-PDR3 -FP | cloning | CCATGCACTAATAAAGACTTGCTATACTC | - |
| 8 | Pro-PDR3-RP | cloning | CCGTTGTTTATTGTGGGAGAC | - |
| 9 | EcoRI-Pro-PDR3 -FP | cloning | CGGAATTCGAACTGTTGAATTAGTATTTAC | *EcoR*I |
| 10 | Pro-PDR3-NcoI-RP | cloning | CATGCCATGGTTTTAATGCTCAAAAAGCCC | *Nco*I |
| 11 | BamHI-AaWRKY1-FP | cloning | TCTCTCTCTAAGCTTGGATCCATGGAAAGTGTTTGTGTTTATG | *BamH*I |
| 12 | AaWRKY1-XbaI-RP | cloning | GATACGAACGAAAGCTCTAGATTAAAATTTGAAATCAAGGTC | *Xba*I |
| 13 | BamHI-AaERF1-FP | cloning | TCTCTCTCTAAGCTTGGATCCATGATGCAAATGCCTTCGTTTG | *BamH*I |
| 14 | AaERF1-XbaI-RP | cloning | GATACGAACGAAAGCTCTAGATTAACCACTAACGGCTTCACTC | *Xba*I |
| 15 | BamHI-AaMYC2-FP | cloning | TCTCTCTCTAAGCTTGGATCCATGACGATGAATATATGGAATT | *BamH*I |
| 16 | AaMYC2-XbaI-RP | cloning | GATACGAACGAAAGCTCTAGATTACCTAGGATCTGACATTC | *Xba*I |
| 17 | BamHI-AaORA-FP | cloning | TCTCTCTCTAAGCTTGGATCCATGTTTGCTACTTGCATTCGCA | *BamH*I |
| 18 | AaORA-XbaI-RP | cloning | GATACGAACGAAAGCTCTAGATCAAAAAAAAAAAAAGTCATCA | *Xba*I |
| 19 | BamHI-AabZIP1-FP | cloning | TCTCTCTCTAAGCTTGGATCCATGAACTACAAGAATTTTGGAA | *BamH*I |
| 20 | AabZIP1-XbaI-RP | cloning | GATACGAACGAAAGCTCTAGATCACCATGGACCGGAAAGTGT | *Xba*I |
| 21 | AaPDR3-QPCR-FP | qPCR | GAGAGGAAAGGCGTTGCGGATT | - |
| 22 | AaPDR3-QPCR-RP | qPCR | GGTGGCTTCTGGATTTGTCATACGG | - |
| 23 | β-actin-FP | qPCR | CCAGGCTGTTCAGTCTCTGTAT | - |
| 24 | β-actin-RP | qPCR | CGCTCGGTAAGGATCTTCATCA | - |
